# A dengue outbreak on a floating village at Cat Ba Island in Vietnam

**DOI:** 10.1186/s12889-015-2235-y

**Published:** 2015-09-22

**Authors:** Thanh Le Viet, Marc Choisy, Juliet E. Bryant, Duoc Vu Trong, Thai Pham Quang, Peter Horby, Hien Nguyen Tran, Huong Tran Thi Kieu, Trung Nguyen Vu, Kinh Nguyen Van, Mai Le Quynh, Heiman FL Wertheim

**Affiliations:** Oxford University Clinical Research Unit Hanoi, Hanoi, Vietnam; MIVEGEC (University of Montpellier, CNRS 5290, IRD 224), Montpellier, France; National Institute of Hygiene and Epidemiology, Hanoi, Vietnam; National Hospital of Tropical Diseases, Hanoi, Vietnam; Nuffield Department of Clinical Medicine, Centre for Tropical Medicine, University of Oxford, Oxford, United Kingdom; Radboudumc, Nijmegen, Netherlands

## Abstract

**Background:**

A dengue outbreak in an ecotourism destination spot in Vietnam, from September to November 2013, impacted a floating village of fishermen on the coastal island of Cat Ba. The outbreak raises questions about how tourism may impact disease spread in rural areas.

**Methods:**

Epidemiological data were obtained from the Hai Phong Preventive Medical Center (PMC), including case histories and residential location from all notified dengue cases from this outbreak. All household addresses were geo-located. Knox test, a spatio-temporal analysis that enables inference dengue clustering constrained by space and time, was performed on the geocoded locations. From the plasma available from two patients, positive for Dengue serotype 3 virus (DENV3), the Envelope (E) gene was sequenced, and their genetic relationships compared to other E sequences in the region.

**Results:**

Of 192 dengue cases, the odds ratio of contracting dengue infections for people living in the floating villages compared to those living on the island was 4.9 (95 % CI: 3.6-6.7). The space-time analyses on 111 geocoded dengue residences found the risk of dengue infection to be the highest within 4 days and a radius of 20 m of a given case. Of the total of ten detected clusters with an excess risk greater than 2, the cluster with the highest number of cases was in the floating village area (24 patients for a total duration of 31 days). Phylogenetic analysis revealed a high homology of the two DENV3 strains (genotype III) from Cat Ba with DENV3 viruses circulating in Hanoi in the same year (99.1 %).

**Conclusions:**

Our study showed that dengue transmission is unlikely to be sustained on Cat Ba Island and that the 2013 epidemic likely originated through introduction of viruses from the mainland, potentially Hanoi. These findings suggest that prevention efforts should be focused on mainland rather than on the island.

**Electronic supplementary material:**

The online version of this article (doi:10.1186/s12889-015-2235-y) contains supplementary material, which is available to authorized users.

## Background

Dengue is a viral vector-borne disease caused by any of four closely related dengue viral serotypes (DENV1, DENV2, DENV3, and DENV4) and transmitted by female *Aedes aegypti* and *Aedes albopictus* mosquitos. Since its reemergence in the 1950’s, dengue has become a major global health problem, with currently more than 390 million people estimated to be at risk in tropical and subtropical regions annually [1–3]. Several dengue outbreaks have been reported from remote tropical islands [4–6], typically caused by introductions of the viruses by viremic travelers who infect local mosquitos. Dengue transmission is mostly influenced by the ecology of the vector population, mosquito-human interaction and the virus serotypes. While no vaccine and specific treatment are available yet, reducing mosquito population density remains the only effective measure of dengue control, both for preventive and responsive purposes. In that context, characterizing and understanding the spatial dynamics of dengue helps design the most efficient vector control program [7].

**Fig. 1:**
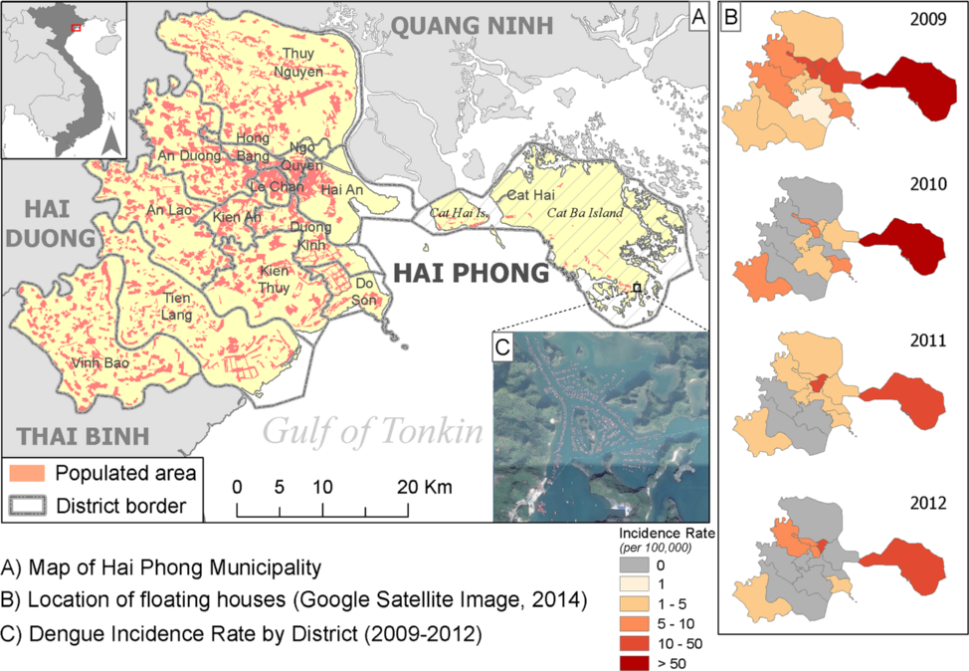
Map of Cat Ba Island, Hai Phong. Cat Hai insular district (including Cat Hai and Cat Ba islands) is highlighted by hatches (**a**); Dengue incidence rate by district (2009–2012) (**b**) and Location of floating houses (**c**)

Cat Ba Island, an insular district of Hai Phong city in northern Vietnam, has a population of approximately 5000 inhabitants, with the majority living in the southern tip of the island. Cat Ba is close to mainland Vietnam (30 km from the center of Hai Phong city, less than two hours by ferry, Fig. 1a) and the island receives more than 500,000 travelers per year [8]. The dengue incidence has historically been found to be much lower in northern Vietnam as compared to southern Vietnam, attributed to climatic differences affecting vector populations [9]. Over the past 5 years, annual dengue cases reported in Hai Phong were low with fewer than 100 cases, except in 2009 when 268 cases were reported [10]. In that year, most dengue cases occurred on Cat Hai and Cat Ba islands (Fig. 1b), concurrent with significant dengue transmission in Hanoi, 100 km away [11]. The outbreak likely resulted from an introduction of dengue viruses from the mainland by travelers [10]. Between September and November 2013, a relatively large dengue outbreak on Cat Ba Island resulted in 192 reported cases. This outbreak was unusual in that most cases were fishermen living in floating houses on the sea (Fig. 1c).

In this study, we characterized the spatial dynamics of the 2013 dengue epidemic in Cat Ba Island and investigated the potential links with the mainland. Conclusions are drawn in terms of local persistence as well as consequences for dengue prevention and control.

## Methods

### Data collection

In Vietnam, dengue cases are detected and reported following the guidelines of Ministry of Health (2011) on surveillance, diagnosis and treatment of dengue. The case definition for suspected dengue infection is based on an acute febrile illness, lasting from 2 to 7 days with a minimum of two clinical symptoms among headache, retro-orbital pain, myalgia, arthralgia, rash, hemorrhagic manifestations, and leucopenia [11, 12]. A single blood sample of a suspected dengue case may be sent for laboratory confirmation by either serology test (IgM ELISA) or molecular methods [11]. For all the dengue-notified cases in Cat Ba, we obtained the following routinely collected information by the dengue control program of Vietnam from the Hai Phong Preventive Medical Center: age, gender, occupation, household address, and date of disease onset. Dengue patient residences were mapped using Garmin GPSMap 78 with accuracy within 20 m (Garmin, USA) to collect GPS coordinates in November in 2013, which was done anonymously.

### Dengue virus characterization

We had access to six positive dengue plasma samples isolated from Cat Ba patients tested by the Department of Virology, National Institute of Hygiene and Epidemiology. Serotypes of the collected samples were verified by Real-Time (Taqman) RT-PCR (Reverse Transcription Polymerase Chain Reaction) at OUCRU research laboratory in Hanoi. There were two samples with sufficient viral RNA (Ct value < 30) to be included for E gene sequencing. The E gene was amplified using 3-fragments conventional PCR according to previously described methods [9, 13, 14]. Amplified fragments with a DNA concentration of at least 50 ng/ml were shipped to Macrogen Inc. (Seoul, South Korea) for sequencing. Genetic relationships of Cat Ba E gene sequences were compared to DENV3 E gene sequences from GenBank and DENV3 sequences isolated from 249 dengue-positive patients admitted to the National Hospital of Tropical Diseases (NHTD) in 2013, Hanoi. The purpose of this comparison was to identify which genotypes the DENV3 sequences from Cat Ba belong to and how closely they are related to other sequences. Therefore, for the DENV3 E sequences from GenBank, we selected only recent isolates since 2000 from southern Vietnam, China, Southeast Asia, Indian subcontinent and South America, with a length of at least 1000 bases.

### Spatio-temporal analyses

We used the Knox test to identify clusters of dengue cases in both space and time, using the onset date to measure time [15, 16]. Space-time interaction resulting from the Knox test is a key metric for infectious diseases [17]. The test counts the numbers of pairs of specific disease cases that are close in both of space and time distance. Specifically, if *n* is the number of dengue cases, the Knox statistic of space-time interactions among collected dengue cases within a given space distance *δ*^*s*^ and time distance *δ*^*t*^ is estimated according to the following equation:

1 $$ Knox=\frac{1}{2}{\displaystyle \sum_{i=1}^n{\displaystyle \sum_{j=1}^n{S}_{ij}}{T}_{ij}} $$

with

2 $$ {S}_{ij}=\left\{\begin{array}{c}\hfill 1,\ \mathrm{if}\ \mathrm{case}\ \left(i\ne j\right)\ \mathrm{and}\ {d}_{ij}\le {\delta}^s\left(\mathrm{meters}\right)\hfill \\ {}\hfill 0,\ \mathrm{otherwise}\hfill \end{array}\right. $$

and

3 $$ {T}_{ij}=\left\{\begin{array}{c}\hfill 1,\ \mathrm{if}\ \mathrm{case}\ \left(\mathrm{i}\ne \mathrm{j}\right)\ \mathrm{and}\ {d}_{ij}\le\ {\delta}^t\ \left(\mathrm{days}\right)\hfill \\ {}\hfill 0,\mathrm{otherwise}\hfill \end{array}\right. $$

*S*_*ij*_ and *T*_*ij*_ are measures of closeness in space and time respectively [18]; *d*_*ij*_ is the distance in space or time between dengue cases *i* and *j*. The cut-off values *δ*^*s*^ and *δ*^*t*^ depend on the nature of the disease transmission and are set by the investigator. In order to reduce the subjective bias in choosing such cut-off values and to account for the minimum of intrinsic incubation period [19], we varied *δ*^*s*^ between 0 and 5000 with a step of 5 m and *δ*^*t*^ between 0 and 60 days with a step of 4 days. At each pair of distances (in space and time), we tested the significance of the Knox statistic against a null distribution generated by Monte Carlo simulations (9999 permutations of onset dates, keeping locations fixed). p-values were estimated as: p-value = R/(9999 + 1); where R is the rank of observed Knox value among the simulated Knox values [16]. Epidemiologically, the ratio between the observed Knox statistic and the mean of simulated Knox statistics can be interpreted as the excess risk (ER) due to the space-time interaction [20, 21]. These ER were visualized as a matrix with temporal distances in rows and spatial distances in columns. The ER(s) were then visually translated to a map of clusters of dengue cases in the area. To group geocoded dengue points into the clusters by space and time, we considered a threshold of space-time distance so that the observed number of pairs of dengue cases should be at least two times higher than the average number of pairs of dengue cases occurred randomly in each clusters, namely excess risk should be greater than 2. The statistical analyses were developed in C++ and R using “Rcpp” package [22] for R version 3.0.2 (R Foundation for Statistical Computing, Vienna, Austria). The source code is available at https://github.com/thanhleviet/knox. Maps were made with ArcMap (ESRI 2011. ArcGIS Desktop: Release 10. Redlands, CA: Environmental Systems Research Institute) and QGIS 2.2.0 (QGIS Development Team, 2013. QGIS Geographic Information System. Open Source Geospatial Foundation Project. http://qgis.osgeo.org)

### Phylogenetic analyses

E gene sequences were aligned using MUSCLE v.3.8.31 [23]. The maximum likelihood (ML) phylogenetic trees were inferred with RAxML (Randomized Axelerated Maximum Likelihood) v. 8.1.17 [24]. GTR + Γ + I was selected as the nucleotide substitution model using jModeltest v.2.1.7 [25]. The statistical robustness and reliability of each node was assessed with 1000 bootstrap replicates. FigTree v.1.4.2 [26] was used to visualize the inferred ML tree.

### Ethics statement

This study was considered as a component of the public health response to the dengue outbreak in Cat Ba in accordance with National Dengue Prevention and Control Program, 2010 – 2015 and part of the responsibility of the National Institute of Hygiene and Epidemiology. Thus, the requirement for ethical review was waived. We ensured data used for this analysis remained anonymous and that the collected GPS locations of dengue household were sufficiently inaccurate (within 20 m of the actual residence) to ensure that patients could not be identified through GPS location. Dengue positive samples from National Hospital of Tropical Diseases were part of the dengue serotype surveillance, which was approved by National Hospital of Tropical Diseases ethics committee. As the surveillance did not collect patient identifiers, written consent was not required.

## Results

Of the 192 reported dengue cases, 154 cases (80 %) were diagnosed based on clinical symptoms, 32 cases (17 %) by IgM ELISA confirmation and 6 cases (3 %) were confirmed as DENV3 by RT-PCR. The patients were from all age groups (median: 37 years [IQR: 27–48]; range: 2–91) and 47 % were male. The dengue cases were mainly from two areas: 123 patients (64 %) lived in Cat Ba town and 69 (36 %) lived in floating houses on the sea, in proximity to Cat Ba town (within 1 km). Accounting for the population size of these two locations (town: 11,000; floating village: 1400), the odds ratio of contracting a dengue infection was 4.9 (95 % CI: 3.6-6.7) for those living in floating houses.

**Fig. 2:**
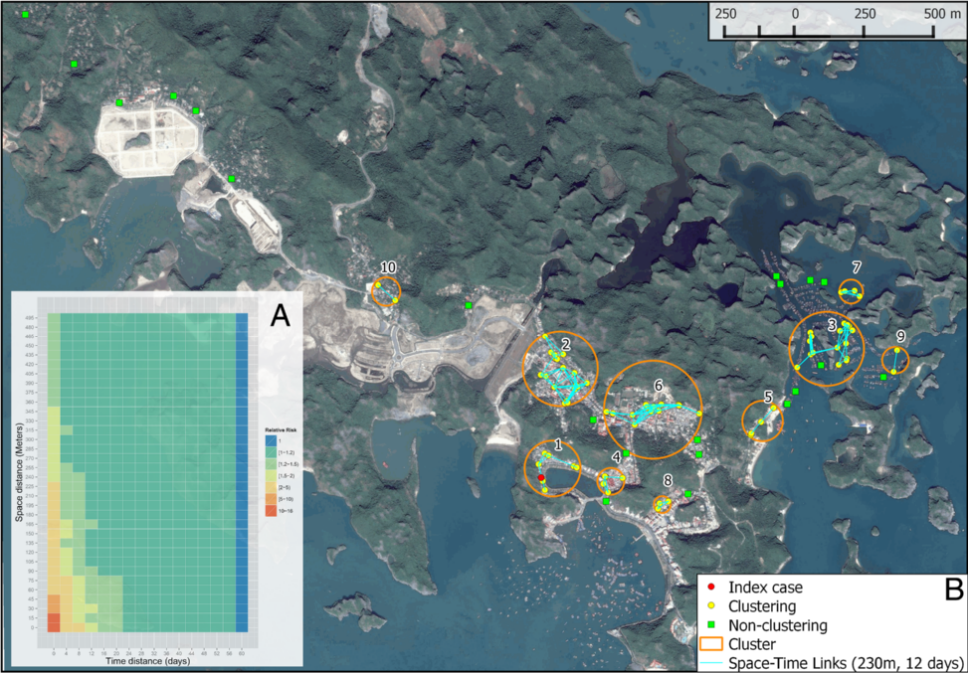
Results of Knox test in a matrix. Rows correspond to space distances by steps of 5 m and columns correspond to time distances by steps of 4 days. Values in each cell are estimated excess risk, colored from the lowest (*blue*) to the highest (*red*) **a**; Map of clusters of dengue cases within a space distance of 230 m and time distance of 12 days **b**; orange circles and numbers represent identified clusters, circle radii only represent cluster extent but not cluster intensity in terms of cases; dots represent location of dengue patients’ household with a fuzzy distance of 20 m around the true position; red dots represent index case, yellow dots represent dengue cases having space-time clustering; green squares represent cases having no space-time clustering; cyan lines represent a possible space-time clustering among cases in each cluster

We were able to collect geographic locations of 111 dengue patients, including the index case (See Additional file 1: Animation of occurrence of dengue cases on Cat Ba Island). Results of the Knox test (Fig. 2a) revealed a significant space-time clustering in which nearby dengue cases occurred at the same period of time (p-value < 0.005). The clustering with highest excess risk was observed at a distance of 20 m and 4 days (ER: 15.8). The risk decreased gradually as time and spatial distances increased. Within a 4-day duration, the risk spread far up to 350 m and shortened in the following days. According to the guidelines of Ministry of Health (MOH), a cluster of dengue is identified when more than two cases are reported within 200 m and within 7 days since the first case reported (3711 QD/BYT). From our matrix results, we found that a distance of 230 m in space and 12 days in time between the dengue cases, giving ER greater than 2, is appropriate for mapping dengue clusters in Cat Ba. The final cluster analysis revealed a total of 10 pairs of 88 (79 %) cases within the selected threshold (Fig. 2b). The clusters were numbered chronologically using the date of the index cases of each cluster. There were seven clusters [1, 2, 4–6, 8, 10] on the mainland of the island, and 3 clusters [3, 7, 9] in the floating village. Cluster 3 had the highest numbers of cases (24 patients) and lasted for 31 days. Characteristics of each cluster are described in Table 1.

**Table 1:** Description of each cluster identified in the study area

Cluster	Onset Distance^a^	Cases	Duration (days)	Time frame	Mean distance (SD)^b^
*1*	0	9	34	Sep/10-Oct/14	75.57(73.92)
*2*	5	18	39	Sep/15-Oct/24	110.6(53.38)
*3*	10	24	31	Sep/20- Oct/21	81.82(63.24)
*4*	11	6	26	Sep/21-Oct/17	81.53(49.31)
*5*	15	4	17	Sep/25-Oct/12	122(19.43)
*6*	22	14	18	Oct/02-Oct/20	114.4(58.77)
*7*	22	6	25	Oct/02-Oct/27	67.27(44.27)
*8*	25	3	12	Oct/05-Oct/17	64.91(25.71)
*9*	29	2	7	Oct/09-Oct/16	157.6(−)
*10*	44	2	6	Oct/24-Oct/30	165.0(−)

^a^Number of days between the onset date of index case of the outbreak and the onset date of the relative index case per cluster

^b^Mean distance (standard deviation) of space-time links of cases per cluster within a distance less than 230 m in space and 12 days in time

**Fig. 3:**
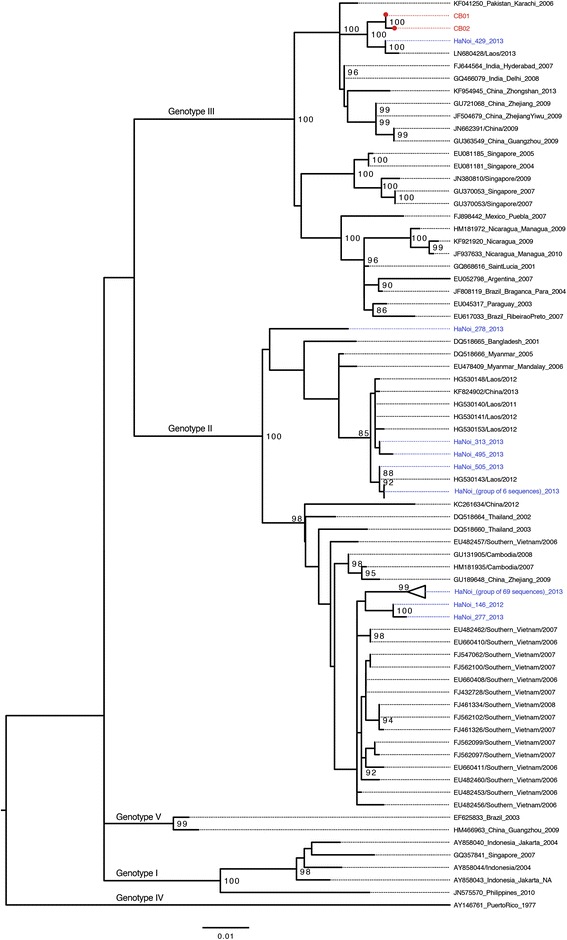
Maximum likelihood phylogenetic tree of DENV3. Red dot circles represent sequences isolated in Cat Ba with tip label named CB01 and CB02, respectively and colored in red. For the sequences obtained from GenBank, tip label includes accession number/isolated location/isolated time. The Hanoi sequences were named following log number of OUCRU-Hanoi laboratory and isolated year, colored in blue. Bootstrap values greater than 80 are shown. Scale bar indicates an evolution distance of 0.01 nucleotide substitutions per site

We were able to sequence two DENV3 serum samples (CB01 and CB02). These two sequences were compared with 82 DENV3 sequences isolated from 249 dengue positive patients admitted to NHTD, and 523 sequences downloaded from GenBank. In the first resultant phylogenetic tree, many sequences from GenBank were identical. For clarity, in the final analysis we selected 62 reference sequences having closest genetic distance to our sequences. Of the reference sequences, 61 strains were selected to represent genotypes I, II, III, V isolated since 2000, and one sequence of genotype IV isolated from Puerto Rico in 1973 that were described elsewhere [27, 28]. The final estimated phylogeny shows that the sequences isolated from NHTD and Cat Ba belong to two main clades of the dengue strains (Fig. 3). Clade 1, belongs to genotype II, and includes 81 strains isolated from NHTD patients and Southeast Asian strains. Clade 2, genotype III, includes CB01, CB02, one strain isolated from NHTD and strains from South America, Indian subcontinent, China, Laos and Singapore. CB01 and CB02 were closely related to the strains isolated in Hanoi and Laos. Both CB01 and CB02 had the highest nucleotide identity to the strain Hanoi_429_2013 (99 % and 99.14 %, respectively).

## Discussion

The dengue outbreak in Cat Ba revealed a clustered distribution of cases with the highest numbers of cases among floating village residents. Accounting for the differences in population sizes, the odds ratio to get infected by dengue in the floating villages as compared to the rest of the island was 4.9 (95 % CI: 3.6-6.7). This was particularly unexpected given that the 2012–2013 entomological surveillance in Cat Ba Island found the densities of both *Aedes aegypti* and *Aedes albopictus* to be lower in floating villages than on the island (no specifics of entomological surveillance methods are provided in this reference and results should be interpreted with caution) [29]. Specifically, in winter (December, 2012), the number of *Aedes aegypti* larvae per 100 houses (larvae index) in the floating villages was 0 while the index was higher in three other locations on the island (residential area: 0.8, tourism area: 0.9, Cat Ba national park: 0.5). In summer (July, 2013), the larvae index of these locations was 0.7, 8.3, 1.6, 3.0, respectively [29]. However, as the population in the floating villages reportedly come from rural districts on the mainland where dengue transmission is relatively rare (oral communication with Cat Ba public health staff), those people were likely more susceptible to dengue infection as compared to the local people living on the island. This may explain why the chance of becoming infected in the floating villages was higher than on the island. In addition, the average distance between cases was less than 100 m and the duration of the occurrence lasted 31 days in the floating villages, indicating that vector control activities implemented in response to the outbreak in the area were likely not effective in reducing dengue transmission. This might be due to poor efficiency of mosquito coils used in the floating houses as compared to malathion 95 % ultra-low volume (MOH guidelines - 973QD/BYT) spray used. ULV was not used in floating houses because of a concern of harming aquatic organisms being farmed beneath the floating houses. In this study, the highest ER was found in a cluster with a short distance (15 m) and just 4 days. This finding is in accordance with previously published results on dengue using Knox statistics, despite different setting in choosing incremental steps [16, 30–32]. For example, Tran et al. [16] used the Knox test over a range of distances in space and time (6500 m and 200 days with steps: 5 m and 1 day) to examine a dengue outbreak of 161 suspected cases in Iracoubo, a small rural municipality in French Guiana. They found a space-time interaction with a relatively high risk within 15 m and 6 days of confirmed dengue cases. When the distances increased from this space-time limit, the risk rapidly decreased. Likewise, in a larger study area, Tartagal city in Argentina, Rotela et al. [32] identified three clusters of 467 suspected dengue fever cases in a range of space and time (8800 m and 109 days with steps: 100 m and 1 day). The first two clusters were found within a short temporal distance (1–3 days) and long spatial distance (100 and 500 m to 2800 m). The third cluster was observed as a second epidemic wave, occurring since day 12 from the index cases, with the same space distance of the second cluster (500 - 2800 m). In principle, these studies found commonly that dengue transmission was very local and constrained by space and time. These patterns were attributed to the blood feeding behavior of the *Aedes aegypti* mosquitos [20, 33]*.* As the *Aedes* mosquitos often take a blood meal on multiple hosts, it increases the chance of infecting dengue viruses to multiple individuals from infectious mosquitos within short spatial and temporal distances (determined by the mosquito flight range). In the case of Cat Ba, this should be true for dengue patients in the floating houses with limited mobility. The particularly narrow clusters of dengue cases observed in Cat Ba Island was likely due to the introduction of a few infected mosquitos infecting multiple individuals, rather than by mobility of viremic humans.

There are two factors that prevent dengue to persist in Cat Ba island: the cold winters and the small size of the human population. The outbreaks usually start with an introduction of dengue viruses by travellers in the warm season, as previously observed [10]. As the island receives a large number of travelers from the mainland each year, this is the most likely route of introduction. This hypothesis is further supported by the dengue surveillance on the island documenting that many domestic travelers from Hanoi and southern provinces (where dengue is endemic) arrived in Cat Ba from June to September in 2013 [29]. While in previous years DENV1 and DENV2 dominantly co-circulated in northern Vietnam, there was a massive replacement of these two serotypes by DENV3 in 2013, as also observed in other places in Southeast Asia [27, 28]. Our phylogenetic results also showed that the two DENV3 isolates in Cat Ba were likely originated from the mainland.

Because the dengue surveillance program following the MOH guidelines (3711 QD/BYT) does not require all dengue cases to be confirmed by PCR, we cannot be certain whether only DENV3 circulated in Cat Ba during the outbreak or also other dengue subtypes. Active serotype surveillance may be considered for small island populations like Cat Ba as it can help in early detection of new serotypes or genotypes to the island before an outbreak starts. This allows for timely information to organize control efforts [5].

Our study had some limitations. First, household locations of dengue patients may not have been the places where the patients acquired infection; it is possible that the persons became infected elsewhere due to their mobility. In addition, as dengue cases are largely unapparent or represent mild symptoms [2], using notified dengue cases only represents only a small fraction of all infections. In the case of Cat Ba outbreak, the population setting is relatively small and there is good access to healthcare, we thus consider that underreporting likely does not reflect a significant proportion of the total number of dengue cases in the floating village in comparison to Cat Ba town. Despite these limitations, it is worth noting that conspicuous clustering was still observed in the data. Second, with 3 % (6/192) of dengue patients confirmed as DENV3 by PCR, it is insufficient to conclude that DENV3 was the dominant cause of the outbreak. However, since DENV3 had reportedly replaced other previous serotypes on mainland [27, 28], the introduction of DENV3 was likely responsible for the large dengue apparent cases in Cat Ba. Third, we only were able to sequence the E gene for two patients, limiting our phylogenetic analysis. However, in such a short timeframe in a single clear outbreak it is unlikely that another strain co-circulated.

## Conclusions

Our study of a dengue outbreak on an island reconfirms that virus transmission is not locally sustained in small populations, and that these epidemics are less frequent and predictable than larger outbreaks occurring in urban areas. Having less frequent epidemics means that the population of susceptibles has more time to reconstruct with a resulting reduced herd immunity, potentially leading to more severe epidemics when it occurs. This, combined with the fact that epidemics are also less predictable, makes island populations particularly at-risk. In such conditions, it appears more efficient to target preventive measures on the mainland and to limit intervention on islands to responsive measures. Efficient serotype surveillances on the mainland can also help predicting the risk of epidemics on the islands to which it is connected.

## Additional file

Additional file 1:
**Animation of occurrence of dengue cases on Cat Ba Island.** The animation movie (file: dengue.avi) shows the occurrence of dengue cases by date during 60 days of the outbreak. (AVI 659 kb)
